# Left Inguino-Scrotal-Perineal Neurofibroma: A Report of a Rare Case and Review of Literature

**DOI:** 10.7759/cureus.103573

**Published:** 2026-02-13

**Authors:** Riyadh Alloqmani, Yazeid I Alrefaey, Mohammed Alghamdi, Jana Hareesi, Salahaldin Lamy, Abeer Nafadi, Abdullah Qashgry

**Affiliations:** 1 Department of Urology, King Abdulaziz Medical City, King Saud bin Abdulaziz University for Health Sciences, Ministry of National Guard Health Affairs, Jeddah, SAU; 2 Department of Pathology, King Abdulaziz Medical City, King Saud bin Abdulaziz University for Health Sciences, Ministry of National Guard Health Affairs, Jeddah, SAU; 3 College of Medicine, King Abdulaziz Medical City, King Saud bin Abdulaziz University for Health Sciences, Ministry of National Guard Health Affairs, Jeddah, SAU

**Keywords:** inguino-scrotal swelling, neurofibroma, paratesticular mass, paratesticular neurofibroma, scrotal mass, solitary neurofibroma

## Abstract

Paratesticular masses arise from structures surrounding the testes, including the spermatic cord, epididymis, vestigial remnants, and tunica vaginalis. These masses are often benign, such as lipomas, leiomyomas, and adenomatoid tumors. Neurofibroma (NF) is a benign tumor of Schwann cell origin, characterized by strong S-100 protein expression. Neurofibromas typically occur in the face, shoulders, arms, periungual regions, and feet; the involvement of the inguino-scrotal region is extremely rare. We report a case of a 66-year-old man with a left inguino-scrotal mass of three years’ duration that progressively enlarged and became painful. After a multidisciplinary review, surgical excision was performed. Histopathology and immunohistochemistry confirmed paratesticular neurofibroma. Neurofibroma should be considered in the differential diagnosis of paratesticular masses, and complete excision remains the treatment of choice.

## Introduction

Neurofibromas (NF) are benign peripheral nerve sheath tumors that may occur as solitary lesions or in association with neurofibromatosis type 1 (NF1), caused by the mutation of the *NF1* tumor-suppressor gene on chromosome 17q11.2 [1]. They are composed of Schwann cells, fibroblasts, perineural cells, and mast cells. Neurofibromas may be localized, diffuse, or plexiform. Solitary neurofibromas can arise throughout the body, most commonly in the neck, thorax, retroperitoneum, and extremities. Paratesticular involvement is exceptionally rare [2-4].

Molecular testing has a limited diagnostic role. Copy number analysis may assist in evaluating atypical neurofibroma or distinguishing it from malignant peripheral nerve sheath tumor (MPNST), which typically demonstrates a complex genomic profile [1].

Most paratesticular masses originate from the epididymis, spermatic cord, or tunica vaginalis and are typically benign. Given the rarity of paratesticular neurofibromas and their nonspecific clinical and imaging features, accurate preoperative diagnosis is challenging, making surgical exploration and definitive histopathological assessment essential for both diagnosis and appropriate management, thereby highlighting the clinical importance of this case.

## Case presentation

A 66-year-old man presented with a left inguino-scrotal mass present for three years, which had progressively enlarged over the past year and recently became painful. The patient denied constitutional symptoms, trauma, prior scrotal surgery, urinary symptoms, flank pain, or hematuria. Physical examination revealed a rubbery, well-circumscribed mass extending from the upper left scrotum to the scrotal neck; both testes were normal and separable.

**Figure 1:**
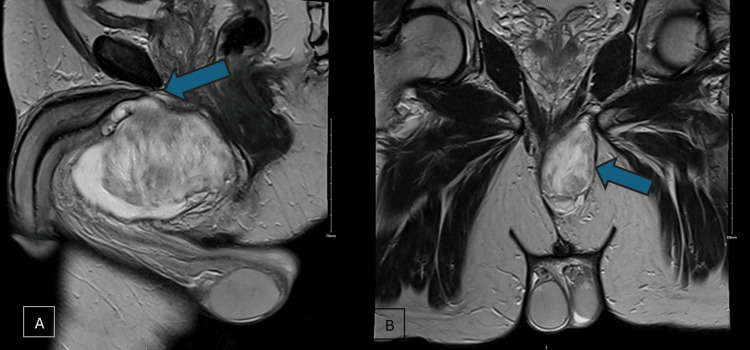
MRI of the scrotum post gadolinium. (A) Sagittal view of the large perineal mass with heterogeneous peripheral enhancement, which is inseparable from the root of the penis (arrowhead). (B) Coronal view of the mass, away from the testis (arrowhead).

Pelvic MRI demonstrated a large, elongated left perineal mass measuring 10.9 × 4.1 × 7.2 cm with heterogeneous peripheral enhancement. The mass was inseparable from the root of the penis, exerting mass effect on the proximal urethra and adjacent structures, prompting a broad differential diagnosis (Figure 1) [5,6].

A multidisciplinary tumor board recommended surgical removal, with spermatic cord liposarcoma considered the leading preoperative differential. A longitudinal left inguino-scrotal incision was made, and the mass was excised en bloc while preserving the spermatic cord and adjacent structures.

Gross evaluation revealed a specimen measuring 11.5 × 7.5 × 3.5 cm with a smooth external surface and a heterogeneous tan-yellow cut surface with focal hemorrhage.

**Figure 2:**
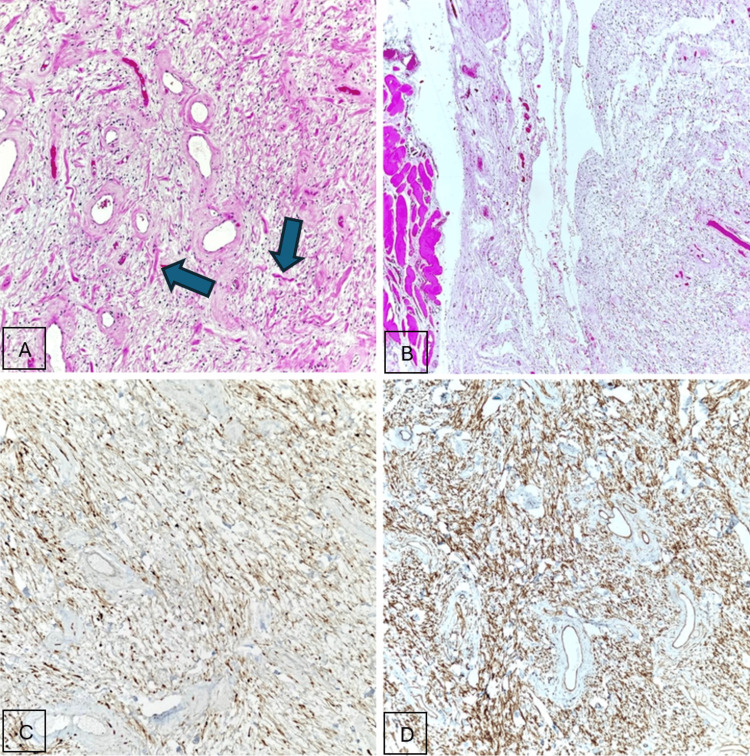
Histologic photomicrographs of the lesion showing (H&E: A, 400×; C, 40×) a spindle-cell neoplasm associated with a variably myxoid to collagenous stroma. Stromal collagen has a characteristic appearance resembling shredded carrots (arrowheads). (B) Neoplastic Schwann cells are represented by bland, spindle-shaped cells with thin, wavy nuclei, highlighted by S-100. (D) Classically, CD34 shows a strong and diffuse latticelike expression pattern in neurofibromas.

Microscopically, the tumor consisted of spindle cells with bland, wavy nuclei and collagen fibrils. Mast cells were readily identifiable, and blood vessels showed hyalinized walls. No Verocay bodies, necrosis, atypical mitoses, or high-grade features were observed. Immunohistochemistry demonstrated strong S-100 positivity and diffuse CD34 expression. Tumor cells were negative for SOX10, SMA, desmin, caldesmon, myogenin, MUC4, CK-pan, calponin, EMA, STAT6, and PR (Figure 2) [1,4,7-9].

The postoperative course was uneventful. At the six-month follow-up, there was no evidence of recurrence.

## Discussion

This case highlights the importance of distinguishing neurofibroma from other paratesticular soft-tissue tumors. Differential considerations include liposarcoma, cellular angiofibroma, and schwannoma. In this case, the immunoprofile, strong S-100 positivity and CD34 expression, with the absence of SOX10, desmin, SMA, EMA, and STAT6, supported the diagnosis of neurofibroma.

Paratesticular neurofibroma is exceptionally rare, with fewer than 20 cases reported in the English literature [4,7-12]. Patients typically present with a painless or mildly painful scrotal mass. MRI provides valuable information regarding lesion size, extent, and the involvement of surrounding structures, facilitating surgical planning.

Histologically, neurofibromas are unencapsulated spindle-cell lesions that are S-100-positive [1,7,8,10-12]. Although SOX10 negativity is uncommon, the combination of morphology and S-100 expression remains diagnostic. Complete surgical excision is curative and carries a low risk of recurrence. Evaluation for NF1 should be considered when there are multiple lesions or systemic features, including café-au-lait spots, Lisch nodules, sphenoidal dysplasia, and optic gliomas [1,7,9].

At the molecular level, most neurofibromas are driven by the inactivation of the *NF1* tumor-suppressor gene on chromosome 17q11.2, which encodes neurofibromin, a negative regulator of RAS-MAPK signaling [1]. The loss of NF1 function leads to the constitutive activation of the RAS pathway, promoting Schwann cell proliferation.

However, molecular testing is rarely performed in neurofibromas. Many published genital cases, including scrotal, spermatic cord, and tunica albuginea neurofibromas, report no clinical signs of NF1 and rely solely on histology and immunohistochemistry for diagnosis (Table 1) [7,9,12-14]. Pediatric cases have also shown classic morphology without the genetic evidence of NF1 [11].

**Table 1:** Expanded summary of reported testicular and paratesticular neurofibroma cases.

Year	Author	Age	Location	Subtype	Tumor Size (cm)	Imaging Findings	Management	Outcome
1939	Schulte et al. [15]	49	Spermatic cord	Typical	Not reported	Not reported	Radical orchiectomy	Not reported
1948	Levant and Chetlin [14]	54	Intratesticular (tunica albuginea)	Typical	Not reported	Not reported	Simple orchiectomy	No recurrence
1975	Jepson [16]	21	Extratesticular (multiple lesions)	Typical	Not reported	Not reported	Debulking (partial excision)	Not reported
1977	Livolsi and Schiff [17]	23	Intratesticular	Typical	Not reported	Not reported	Radical orchiectomy	No recurrence
1982	Yamamoto et al. [7]	8	Extratesticular	Typical	5.0 × 1.5 × 1.5	Not reported	Complete excision (testis-sparing)	No recurrence (one year)
1990	Yoshimura et al. [3]	41	Extratesticular	Typical	3.0 × 3.5 × 5.0	Not reported	Complete excision (testis-sparing)	No recurrence (one year)
1993	Issa et al. [8]	77	Extratesticular	Typical	15 × 4.5 × 3.0	Ultrasound (US): solid scrotal mass	Complete excision (testis-sparing)	No recurrence (one year)
2002	Deliveliotis et al. [4]	74	Spermatic cord	Typical	4.0 × 4.0 × 1.0	Not reported	Radical orchiectomy	No recurrence (four months)
2004	Milathianakis et al. [11]	86	Spermatic cord	Typical	Not reported	Not reported	Complete excision (testis-sparing)	No recurrence (19 months)
2004	Türkyilmaz et al. [12]	11	Extratesticular	Typical	Not reported	Not reported	Complete excision (testis-sparing)	No recurrence (one year)
2008	Erdemir et al. [18]	45	Extratesticular	Typical	3.0 × 2.0 × 1.0	US: hypoechoic; CT: ~3 cm mass	Complete excision (testis-sparing)	No recurrence
2012	Hosseini et al. [9]	45	Extratesticular	Typical	9.0 × 4.5	US: hypoechoic; CT: 9 cm mass	Complete excision (testis-sparing)	No recurrence (five months)
2015	Boto et al. [10]	38	Spermatic cord	Typical	≈3.0-4.0	MRI: T2 hyperintense mass	Complete excision (testis-sparing)	No recurrence
2016	Chandrashekar et al. [19]	32	Extratesticular (inguino-scrotal)	Typical	10 × 20	Not reported	Complete excision (testis-sparing)	Not reported
2021	Thorat and Khachane [20]	77	Extratesticular	Typical	20 × 9.0	US: heterogeneous hypoechoic mass	Complete excision (testis-sparing)	No recurrence (one month)
2024	Jaber et al. [21]	6	Extratesticular	Plexiform	≈5.5 × 3.0	MRI: T2 hyperintense; target sign	Complete excision (testis-sparing)	No recurrence (one year)

The WHO classification notes that molecular testing is not required for diagnosing conventional neurofibroma but may be helpful in atypical lesions. Atypical neurofibromas often show *CDKN2A*/*CDKN2B* deletions, while MPNSTs exhibit complex genomic alterations “including TP53 and PRC2 component loss” [1]. Older reports of genital neurofibroma predated modern molecular testing, but authors occasionally noted that cytogenetic studies may help differentiate neurofibroma from low-grade sarcoma [7].

Malignant transformation is exceedingly rare in solitary sporadic neurofibroma and is primarily associated with plexiform neurofibroma in patients with NF1. No malignant transformation has been reported in genital neurofibromas without NF1 [8-12]. For tumors with benign morphology, low mitotic activity, and classic immunophenotype, routine molecular analysis is unlikely to alter management. Nonetheless, in large, rapidly growing, or histologically atypical paratesticular tumors, targeted molecular studies (NF1, *CDKN2A*/*CDKN2B*, and copy number profiling) may help exclude early MPNST.

In our patient, the benign histologic features, conventional immunoprofile, absence of NF1 stigmata, and uneventful postoperative course strongly support a diagnosis of a sporadic neurofibroma, with no indication for additional molecular workup.

## Conclusions

Paratesticular neurofibroma is a rare benign tumor requiring thorough clinical, radiologic, and histopathological assessment to ensure accurate diagnosis. Complete surgical excision offers an optimal outcome, and short-term follow-up is recommended to monitor for recurrence. Although recurrence is extremely rare, routine long-term surveillance is not mandated by the WHO criteria. The awareness of this rare entity helps prevent misdiagnosis and avoid unnecessary radical surgical interventions.
